# Prenatal Diagnosis and Pathology of Laryngeal Atresia in Congenital High Airway Obstruction Syndrome

**DOI:** 10.1155/2012/616905

**Published:** 2012-12-24

**Authors:** Piya Chaemsaithong, Tharintorn Chansoon, Boonsri Chanrachakul, Suchin Worawichawong, Sansanee Wongwaisayawan, Patama Promsonthi

**Affiliations:** ^1^Department of Obstetrics and Gynaecology, Ramathibodi Hospital, Faculty of Medicine, Mahidol University, 270 RamaVI Road, Phayathai, Rajthevee, Bangkok 10400, Thailand; ^2^Department of Pathology, Ramathibodi Hospital, Faculty of Medicine, Mahidol University, 270 RamaVI Road, Phayathai, Rajthevee, Bangkok 10400, Thailand; ^3^Women Center, Bumrungrad International Hospital, 33 Sukhumvit 3, Wattana, Bangkok 10110, Thailand

## Abstract

Congenital high airway obstruction syndrome is a rare but life-threatening condition. Therefore, prenatal diagnosis is important. The obstruction can be due to laryngeal/tracheal atresia or external compression. While a differential diagnosis with congenital cystic adenomatoid malformation (CCAM) type III may be difficult, it is still possible with ultrasonography. In this study, we report a case of bilateral echogenic lungs with hydrops fetalis. After the prenatal diagnosis of laryngeal atresia, the couple opted to have an elective termination of pregnancy performed at 20 weeks of gestation. The diagnosis was confirmed by a complete pathological examination.

## 1. Introduction

Congenital atresia of the larynx is a rare abnormality of upper airway obstruction. For such a condition, prenatal diagnosis is difficult. Nevertheless, the diagnosis is crucial because affected fetuses face a high risk of demise. Sonographic findings showed increased lung echogenicity and size coexisting with fetal ascites. However, the findings may not always be typical. We reported a 20-week-old male fetus with congenital laryngeal atresia diagnosed prenatally by the findings of fetal hydrops, hyperechogenic lungs and other abnormalities without the fluid-filled trachea. After the elective termination of pregnancy, the diagnosis was confirmed by a complete pathological examination. 

## 2. Case Report

**Figure 1:**
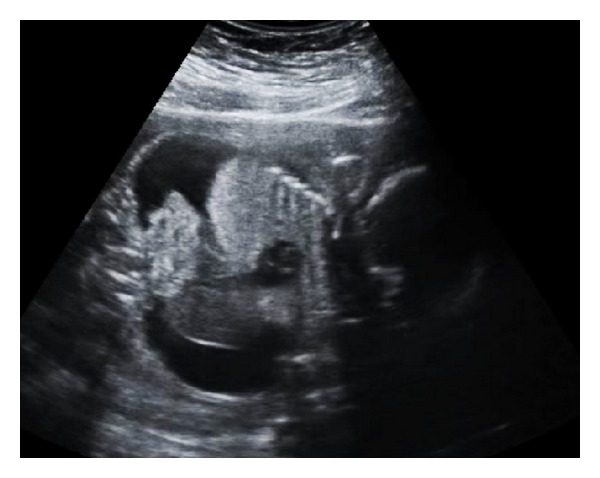
Coronal scan through the fetal chest and abdomen revealed bilateral echogenic lungs and ascites.

**Figure 2:**
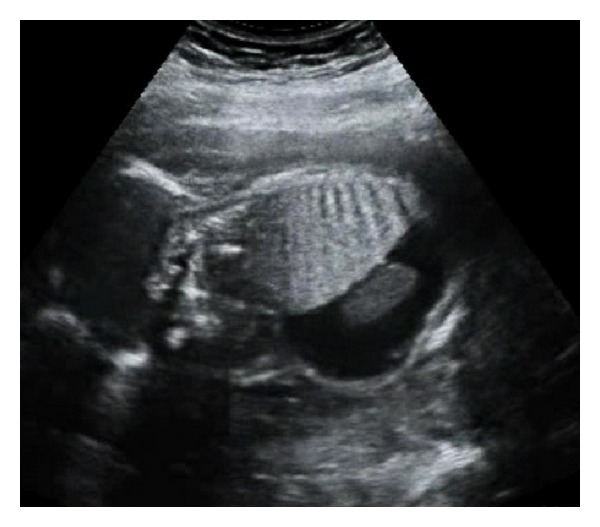
Sagittal view: note the inversion of the diaphragm.

**Figure 3:**
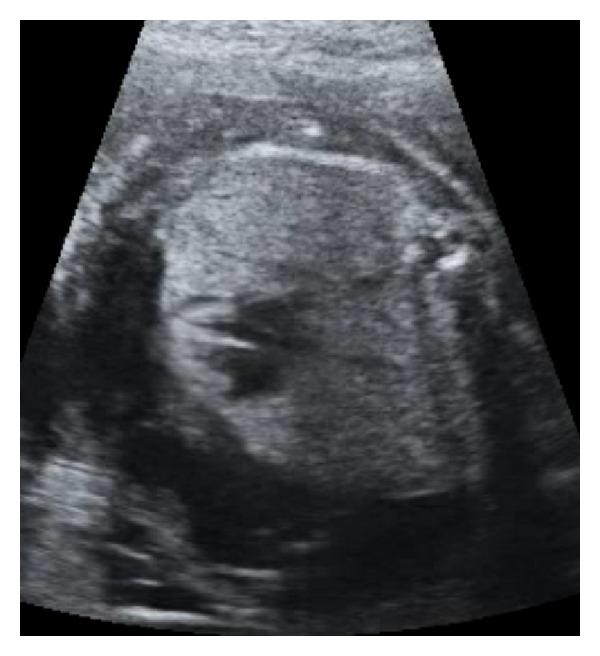
Axial scan of the fetal chest: the heart was squeezed by the hyperechoic lungs.

A 29-years-old, gravida 1, woman was referred to our perinatal unit at 20 weeks of gestation on suspicion of fetal anomaly. Her family history was noncontributory. Physical examination revealed a 20-week-sized uterus with audible fetal heart sound. Transabdominal ultrasound showed oligohydramnios and fetal hydrops with marked ascites, generalized skin edema and placentomegaly. The fetal chest circumference was larger than 95th percentile of 20 weeks gestation. Both lungs became severely enlarged and highly hyperechoic (Figure 1). Inversion of the diaphragm was also noted (Figure 2). Fetal lung volume calculated by two-dimensional sonography was 21 mL, which was more than the 99th percentile for the gestational age (7.03 mL) [1]. Neither pleural nor pericardial effusion was found. The heart was squeezed by the echogenic lungs (Figure 3), albeit structurally normal. The dilated trachea cannot be demonstrated. No other anomalies were noticed. The diagnosis of congenital high airway obstruction syndrome (CHAOS) was made. After the counseling session, the parents elected to terminate the pregnancy, and an autopsy was performed. 

## 3. Pathology

**Figure 4:**
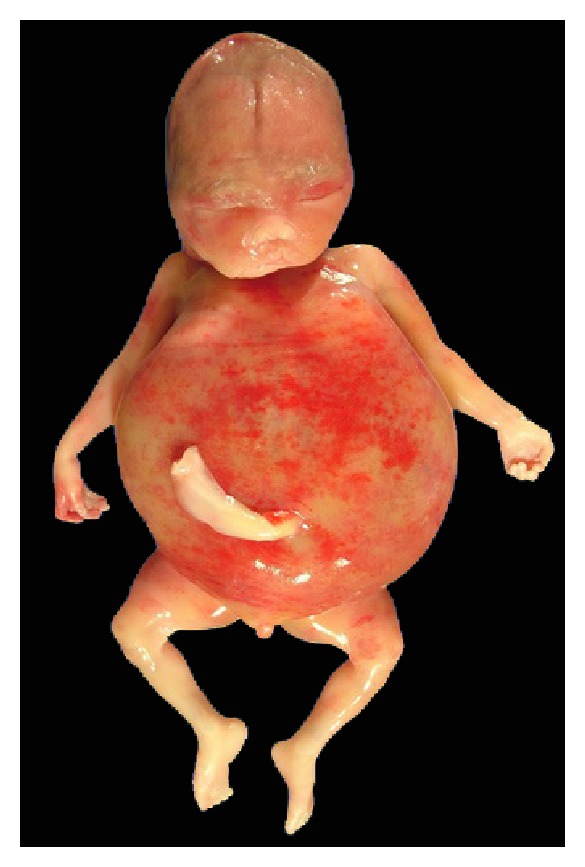
Postabortion: generalized edema with markedly distended abdomen.

**Figure 5:**
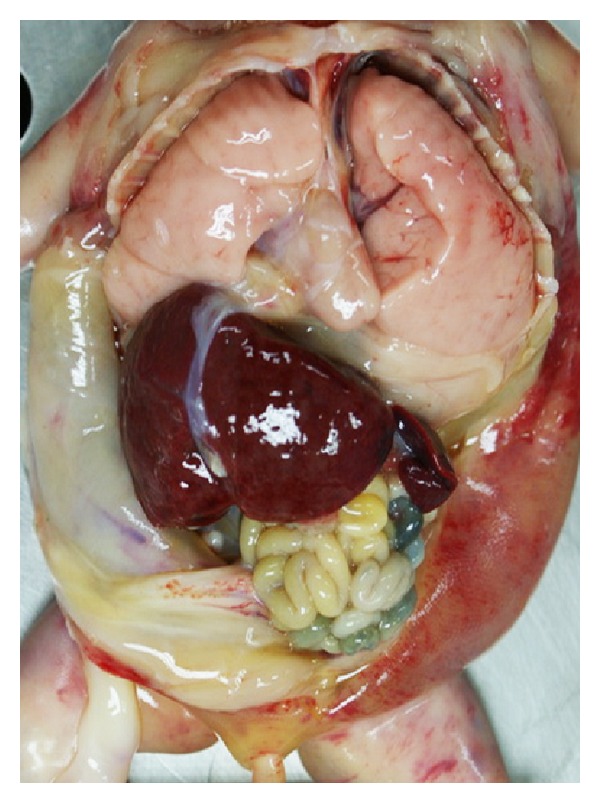
Gross appearance of thorax and abdomen show enlarged both lungs with costal impression on the external surface.

**Figure 6:**
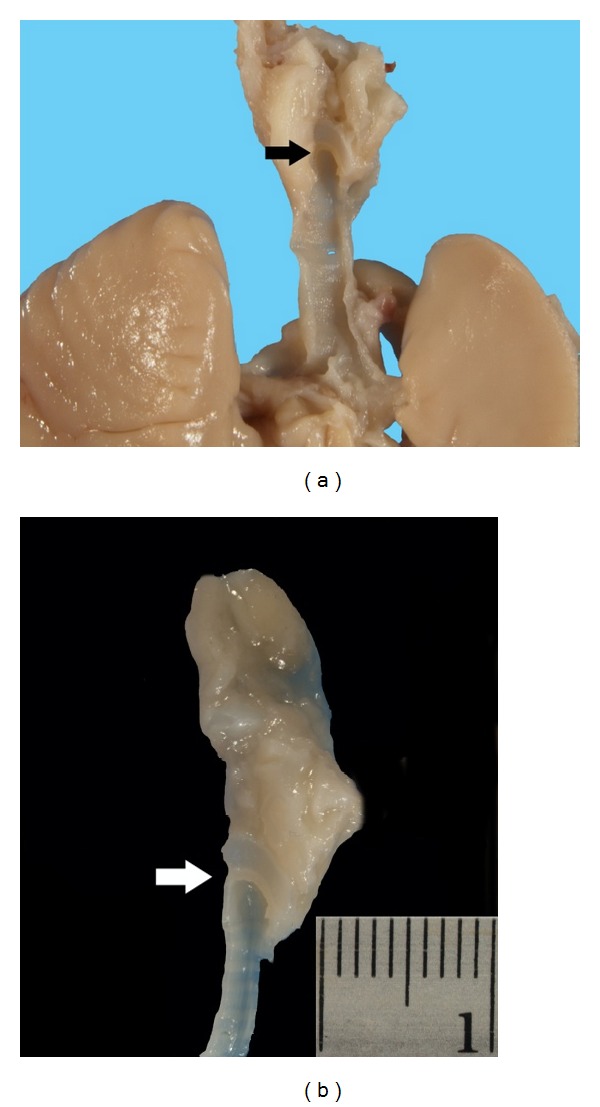
(a) Bisection of the neck block demonstrates complete obstruction of the larynx (arrow). (b) Sagittal view of the larynx and trachea demonstrates atresia at subglottic level (arrow).

The aborted fetus weighed 450 g and had a distended thorax and abdomen and deformation both ear lobules. Postmortem examination revealed generalized edema and markedly distended abdomen compatible with hydrops fetalis (Figure 4). The abdominal cavity showed a large amount of clear yellow fluid and an incomplete rotation of the intestine. An intrathoracic examination showed the enlargement of the right and the left lungs, which weighed 7.4 and 8.7 grams, respectively. The right lung had 3 lobes, while the left had one lobe. Costal impressions were observed on the external surface of both lungs (Figure 5). Microscopic examination revealed thinning of the epithelium at the periphery of the lungs with the development of intraacinar capillaries. The larynx showed complete obstruction at the infraglottic level caused by the overgrowth of the cricoid cartilage (Figure 6). Neither tracheoesophageal fistula nor esophageal atresia was observed. Autopsy findings were compatible with laryngeal atresia type II described by Smith and Bain in 1965 [2]. The cord blood cytogenetic study revealed 46, XY karyotype.

## 4. Discussion

Congenital high airway obstruction syndrome (CHAOS) is a rare and usually lethal abnormality. Three possible presentations include (1) complete laryngeal atresia without an esophageal fistula, (2) complete laryngeal atresia with a trachea-esophageal fistula, and (3) near-complete high upper airway obstruction [3]. Many etiologies were proposed including laryngeal or tracheal webs, laryngeal cysts, tracheal atresia, subglottic stenosis or atresia, laryngeal or tracheal agenesis. However, laryngeal atresia appears to be the most frequent cause. Prenatal diagnosis of CHAOS was possible as early as 15 weeks of gestation [4]. The sonographic findings of CHAOS include increased in lung size and echogenicity, fluid-filled, dilated trachea, fetal hydrops, and polyhydramnios. Prenatal identification of level of atresia is difficult. However, Kalache et al. were the first to describe the application of color and spectral Doppler techniques to localize the level of atresia in fetuses with CHAOS [5]. Until recently, there have been nearly 40 cases of prenatally diagnosed laryngeal atresia including our case [6, 7]. 

Laryngeal atresia may be associated with other structural and genetic abnormalities [8], such as left persistent superior vena cava, single umbilical artery, abnormal fingers and toes, esophageal atresia, or renal agenesis [5, 9]. Partial trisomy 9 and 16, chromosome 5p deletion, and 22q11.2 deletion were also reported in association [10–12]. It is important to recognize the syndromes associated with laryngeal atresia in order to provide appropriate counseling for future pregnancies. 

Although laryngeal atresia is usually lethal, it has recently become possible to bypass the airway obstruction and establish adequate ventilation by the EXIT procedure (Ex Utero Intrapartum Treatment) or fetoscopic laser decompression, while the fetus is still connected to the placenta. Several successful cases have been reported [10, 13–18]. Even with the presence of hydrops fetalis, successful fetal intervention by tracheostomy and EXIT procedure were recorded [19, 20].

In this case, we observed enlarged bilateral echogenic lung masses, inversion of the diaphragm with nonimmune hydrops fetalis. Oligohydramnios can be present in the context of hydrops due to the impairment of renal function. The findings were difficult to differential with congenital cystic adenomatoid malformation (CCAM) type III. However, bilateral CCAM was very rare; therefore, the diagnosis of CHAOS was considered. Moreover, our case had distinct abnormalities, including deformed ears, a single lobe in the left lung, and intestinal malrotation. Lastly, our case did not have an enlarged fluid-filled trachea, a unique sonographic feature, although the laryngeal stenosis without fistula was confirmed by the pathological examination.
